# Expert Consensus on Components of an Evidence‐Based Framework to Facilitate Self‐Employment for Persons With Disabilities: A Delphi Study

**DOI:** 10.1155/oti/4109837

**Published:** 2026-05-21

**Authors:** Luther Lebogang Monareng, Shaheed Mogammad Soeker, Deshini Naidoo

**Affiliations:** ^1^ Occupational Therapy Department, University of KwaZulu-Natal, Durban, South Africa, ukzn.ac.za; ^2^ Occupational Therapy Department, University of the Western Cape, Bellville, South Africa, uwc.ac.za

**Keywords:** entrepreneurship, inclusion, livelihood, microenterprises, rehabilitation, vocation

## Abstract

**Background:**

In light of the global unemployment crisis, there is a need for research on nonconventional work, including self‐employment, an alternative placement option for persons with disabilities.

**Objective:**

The objective of the study is to establish consensus on the components of an evidence‐based framework to facilitate self‐employment for persons with disabilities.

**Methods:**

End users, service providers and policy actors participated in this Delphi study. Relevant data was collected online and analysed thematically, descriptively and using frequencies.

**Results:**

The response rates were *n* = 19/21 (91%) in Round 1, *n* = 16/19 (84%) in Round 2 and *n* = 15/16 (94%) in Round 3. The main findings of Round 1, through an open‐ended question, were (i) an ideal tool, considerations and interventions related to entrepreneurial skills for persons with disabilities and (ii) expected roles and capacities of persons with disabilities. Through the 5‐point Likert scale evaluations in Round 2, the participants identified critical components of the framework, including (i) *key features*, (ii) *legislation* that informs disability rights, (iii) involved *key role players*, (iv) *characteristics, roles and duties of persons with disabilities* and (v) the ideal tool’s *facilitation steps*. In the final round, the participants agreed on the content of the framework’s user’s guide document to include the following: (i) *purpose of the tool*, (ii) *background of the tool*, (iii) *how to use the tool* and (iv) *tool supporting resources*.

**Conclusion:**

Consensus on the components of a framework was achieved; experts regarded the framework as being nonprescriptive, prioritising practicality, accessibility and sustainability within and beyond the South African context. Participants proposed measures like a user’s guide to support framework operationalisation.

**Summary**

In this research, the term ‘tool’ referred to a range of resources, including training, education programmes, guidelines or evidence‐based frameworks. This approach was meant to simplify and accommodate the diverse and heterogeneous sample, facilitating their meaningful participation, that is, to ensure that no critical components are overlooked during the study. ‘Tool’ is used when reflecting participants’ wording and ‘framework’ to refer to the conceptualised output.

## 1. Background

The global unemployment crisis disproportionately impacts vulnerable populations, particularly the 15% of people with disabilities, who face significant barriers to conventional employment [1, 2]. A key contributing factor is the limited availability of suitable work opportunities for all capable individuals, with persons with disabilities competing alongside others for scarce opportunities [3, 4]. Self‐employment offers a potential solution but lacks profession‐specific, transparent frameworks to support persons with disabilities effectively [5]. The neglect of self‐employment persons with disabilities in the literature is often attributed to its complexity and scarcity of research offering tailored, practical solutions [6, 7]. This research contributes towards addressing this neglect through establishing consensus on components of an evidence‐based framework to facilitate self‐employment for persons with disabilities, with a specific focus on microenterprises as one form of self‐employment relevant to the South African context, by using the Delphi technique with subject experts (participants).

Prolonged absence from employment following injury, an occurrence frequently experienced in adulthood [8], represents a significant contributor to the global unemployment crisis. It is also important to note that employment challenges can also arise for individuals with disabilities who have never entered the workforce. This trend persists irrespective of the nature of the injury or disability, encompassing physical impairments, traumatic brain injury or mental illness [9, 10]. While professionals such as occupational therapists facilitate return to work through vocational rehabilitation services [11, 12], barriers persist when conventional employment is unavailable or vocational rehabilitation programmes lack tailored approaches. Consequently, affected individuals or persons with disabilities remain unemployed in conventional work, making self‐employment (entrepreneurship) a possible alternative. Although persons with disabilities prefer this type of vocation [13], it lacks robust research, as stated by Bhardwaj et al. [14], p. 8, that ‘Underdog entrepreneurship literature has still remained at an embryonic stage’.

The research gap in self‐employment for persons with disabilities is attributed to the lack of sufficient interest from key role players, including researchers and those offering vocational services [6], potentially being discouraged by time‐intensive processes, such as securing microenterprise financing [15]. In addition, key role players, such as occupational therapists, have contributed to this gap through inconsistent or nonstandardised vocational terminologies, which hamper the comparability and accumulation of research in this area [16–18]. Sodhi and Dwivedi [6] advocate for future research to examine the growth and scale‐up stages of businesses owned by persons with disabilities. These authors emphasise the role of government support and nonprofit organisations (NPOs) in providing business customised training for efficiency and sustainability. Additionally, researchers endorse leveraging peer insights or learning for mentorship in business developments [19]. There is a need to evaluate the sustainability of microenterprises operated by persons with disabilities [15].

The multifaceted aspects of work, indicated above, suggest that there is no quick‐fix solution [15] or panacea to the global unemployment crisis. It is also evident that addressing the paucity of literature in self‐employment for persons with disabilities [6] requires more than a single or simplistic approach, necessitating strategic and systematic efforts. This study is aimed at establishing consensus on the components of an evidence‐based framework to facilitate self‐employment for persons with disabilities. As such, this research, part of a multiphase PhD project, included a first‐phase scoping review that mapped out literature on evidence‐based frameworks, yielding results suited for high‐income countries. The second phase engaged key role players, including persons with disabilities (end users), who unanimously proposed components for a South African context‐sensitive, evidence‐based framework to facilitate self‐employment for persons with disabilities. This research paper is built on the previous phases through the multiround Delphi technique, where subject matter experts worked collaboratively to develop the framework components further. The experts responded to open‐ended questions in Round 1, rating synthesised components using a 5‐point Likert scale in Round 2, and evaluating a user’s guide with binary agree or disagree options in Round 3. The final deliverable of the PhD project involves translating theoretical consensus into practical application by operationalising the framework for role players supporting self‐employment initiatives among persons with disabilities, thereby enabling income generation, independence and the pursuit of a meaningful life. Moreover, findings indicate that evidence‐based methods enhance decision‐making in professions, including healthcare [20]. Because the current study is aimed at contributing towards developing justified components of an evidence‐based framework, a method grounded in expert judgement is required. The Delphi technique is well‐established as an evidence‐based method for achieving expert consensus, making it appropriate for refining the framework components developed in earlier phases of the PhD project. Although time‐intensive, the intended framework offers benefits such as improved sustainability for microenterprises and increased earnings for owners [15] owing to its evidence‐based and consensus‐driven design.

Disclaimer: Self‐employment in microenterprises may not be appropriate or desirable for all individuals with disabilities. This should be noted by all those who are involved in this field.

## 2. Method

This study used a Delphi technique. The first round of the Delphi survey builds on previous PhD phases, one and two. The Delphi technique was used to engage experts (knowledgeable participants) in this field, aiming to achieve consensus on the components of an evidence‐based framework to support self‐employment for persons with disabilities in a South African context. The method section is outlined under the following subheadings: (i) Design, (ii) Identification and Recruitment of Participants (Sampling), (iii) Procedure (Rounds 1, 2 and 3), (iv) Data Management, (v) Data Analysis, (vi) Ethical Consideration and (vii) Data Dissemination.

### 2.1. Design

To the researchers’ knowledge, there is no existing or published contextual framework in self‐employment for persons with disability; therefore, the Delphi technique’s sequential nature and multiple rounds were suitable for this research [21, 22]. Involving a heterogeneous group of participants facilitated diverse perspectives on the same subject, enhancing understanding through consensus building [23]. Moreover, the Delphi technique allowed for collecting unique data from each individual without the influence of dominant participants or group pressure dynamics, which can occur in techniques like focus groups [24]. Overall, the Delphi technique involved identifying and recruiting group members and constructing and distributing Rounds 1, 2 and 3. Ultimately, there was an achievement of group consensus on the framework components for self‐employment for persons with disability.

### 2.2. Identification and Recruitment of Participants (Sampling)

Delphi studies have no agreed‐upon sample size in the literature [22, 25]. This research is aimed for at least two participants per expert category to ensure balanced representation. Participants’ categories and inclusion criteria aligned with the project’s previous two phases are outlined in Table 1. The recruitment targeted participants from within and beyond South Africa, consistent with Phase 1 (where the researcher identified and invited authors from scoping review articles) and Phase 2 (where end users, service providers and policy actors were identified and invited as potential participants) of the PhD project. Such an approach implies that individuals known to meet the inclusion criteria, as outlined in Table 1, were invited.

**Table 1:** Participants’ categories and inclusion criteria.

Expert (participants) category	Inclusion criteria
Persons with disabilities	○ Meet the 18–65 years South African working age
	○ Have been running a microenterprise for at least 3 years and making a living from it [26]
	○ Have a disability

Occupational therapist	○ Have 3 or more years of work experience
	○ Have knowledge about vocational rehabilitation
	○ Occupational therapists who are clinicians, based at Learners with

Persons with disabilities, organisations and representatives	○ Working for at least 3 years
	○ With knowledge about self‐employment in microenterprises for persons with disabilities

Government department representatives, for example, from the Department of Labour and Employment	○ Working for at least 3 years
	○ With knowledge about self‐employment in microenterprises for persons with disabilities

A multisampling approach, integrating purposive and snowball sampling, was used to identify and recruit these suitable participants. The recruitment email included an information sheet, consent form, participant expectations, an ethics clearance letter and the Delphi provisional timelines. Initial recruitment efforts involved sending bulk emails to two associations and/or interest groups, Occupational Therapy Association of South Africa (OTASA) and Rural Rehab South Africa (RuReSA), through their chairpersons. However, these efforts elicited no response. Subsequent individualised email invitations were sent to over 45 potential participants. However, more than 20 recipients did not respond or declined to participate due to other commitments or perceived unsuitability. Ultimately, 21 participants agreed to participate.

### 2.3. Procedure

After recruitment, the data collection process followed three sequential Delphi rounds, summarised in Table 2. The entire process, from recruitment to Round 3 (the final round), spanned 5 months (July to December 2024). All communication occurred through email and data were gathered using an online self‐administered questionnaire created with Microsoft 365 Forms. Rigour and trustworthiness [27, 28] were enhanced through an iterative process involving the corresponding author and two coauthors. Additionally, all questionnaires were piloted with two individuals, each holding a master’s degree and enrolled on PhD studies. Enhancement of the questionnaires, stemming from the iterative process and pilot, included the following: (i) addressing technical issues, such as ensuring all fields are mandatory before participants proceed to the next section of the questionnaire; (ii) rephrasing terminology for clarity and accuracy, for example, changing ‘persons living with disabilities’ to ‘persons with disabilities’ and (iii) streamlining question relevance, which involved removing nonessential sections to reduce questionnaire completion time and encourage expert participation, for example, omitting redundant information sheets in subsequent rounds.

**Table 2:** Summary of the Delphi technique rounds.

Recruitment and panel selection	○ Purposive and snowball sampling through email
	○ Stimulus package draft informed by PhD phases one and two findings
Round 1	○ Consent received and demographic information collected
	○ Data collection using a stimulus package
	○ Thematic, descriptive and frequency data analysis
Round 2	○ Consensus on components of an evidence‐based framework (informed by Round 1)
	○ Thematic, descriptive and frequency data analysis
Round 3 (final)	○ Further consensus and confirmation of Round 2 findings
	○ Evaluating a user’s guide with binary agree or disagree options
	○ Thematic and frequency data analysis

#### 2.3.1. Round 1

The aim is for participants to respond to open‐ended questions based on a questionnaire (stimulus package) informed by findings from phases one and two of the PhD project.

The content of Round 1’s questionnaire (stimulus package), detailed below, was informed by the PhD project’s findings from phases one and two. This round spanned 2 weeks, with reminder emails sent at the midpoint (1 week) and on the final day to increase participation and optimise response rates. Individual emails about the first round were sent to each participant who expressed interest, including the following key content: an estimated completion time of approximately 30 min, a submission deadline within 2 weeks, relevant documents (such as an information sheet and the research’s ethical clearance certificate) and a questionnaire link for an online self‐administered questionnaire.

In addition to the participants’ demographics, the online questionnaire prompted the participants to answer open‐ended questions by providing input on the following: (i) self‐employment or entrepreneurial opportunities (small businesses) persons with disabilities successfully engage in; (ii) participants’ awareness of tools (e.g., training/education programme, guidelines and evidence‐based framework) used to facilitate self‐employment for persons with disabilities in South Africa, Africa or in any part of the world; (iii) components to include or consider in an ideal tool (training/education programme, guidelines and evidence‐based framework) to successfully facilitate self‐employment for persons with disabilities; (iv) interventions related to entrepreneurial skills for persons with disabilities (including specific interventions and processes to be applied); and (v) the capacity in which persons with disabilities (individually and collectively) should participate in self‐employment or entrepreneurial opportunities (small businesses).

#### 2.3.2. Round 2

The aim is for participants to reach consensus by rating synthesised components of the evidence‐based framework (derived from Round 1) using a 5‐point Likert scale.

Round 2 is built upon the cumulative insights from Round 1. Clear instructions and expectations were emailed to participants, including the due date and a link to the online questionnaire. The questionnaire’s main structure consisted of Sections A–E, as detailed in Table 3.

**Table 3:** Round 2 questionnaire’s main structure.

Section	Description
A. Key features or characteristics of the ideal tool	For consistency when used, the ideal tool (training/education programme, guidelines, evidence‐based framework) should have and stick to key features or characteristics.
B. Legal framework and laws	The ideal tool (training/education programme, guidelines, evidence‐based framework) that facilitates self‐employment should consider relevant laws—international legal frameworks or laws and national (South African) legal frameworks or laws.
C. Key role players in using the ideal tool	The nonprescriptive ideal tool (training/education programme, guidelines, evidence‐based framework) to succeed, credible and reliable various key role players from different settings ranging from community to national and international level must work collaboratively, that is, not in silos when rendering services to persons with disabilities in self‐employment.
D. Persons with disabilities’ characteristics, roles and duties	At the centre of the nonprescriptive ideal tool (training/education programme, guidelines, evidence‐based framework) should be capable persons with disabilities in self‐employment with clear roles and duties.
E. Ideal tool facilitation steps (processes)	Steps (processes) to be followed when mobilising necessary resources and facilitating self‐employment for persons with disabilities using the nonprescriptive ideal tool (training/education programme, guidelines, evidence‐based framework)
	• Step 1 (preparing the ground or conducting a situation analysis)
	• Step 2 (intervention or bridging the gap)
	• Step 3 (placement or setting up the microenterprise)
	• Step 4 (carryover and stabilising the microenterprise)
	In order to have the graded four steps to succeed, there is a need for an individual (case manager/facilitator) or institution to oversee and/or facilitate these steps.

For each section outlined in Table 3, participants were requested to critique the content by (i) rating items using a standardised 5‐point Likert scale. For ratings below 4 (80%), participants were required to suggest improvements; (ii) estimating the duration of the tool and processes by selecting options ranging from 3 months to over a year for each item and (iii) offering additional comments in the provided space for each section. Round 2 mirrored Round 1’s timeline, that is, lasting 2 weeks, with reminder emails sent at the midpoint (1 week) and on the final due date to increase response rates.

#### 2.3.3. Round 3 (Final Round)

The aim is for participants to evaluate a draft user’s guide using binary agree or disagree options to confirm consensus on its content and usability.

In Round 3, the framework components which reached consensus in Round 2 were refined and reorganised to inform a user’s guide that is user‐friendly and easy to follow for case managers or facilitators who will operationalise the final tool. Specifically, the participants reviewed and confirmed the user’s guide content. They confirmed their option by selecting ‘agree’ or ‘disagree’, with spaces provided for additional comments. Table 4 outlines the main structure of the Round 3 questionnaire. The logistics for Round 3 aligned with those of previous rounds.

**Table 4:** Round 3 questionnaire’s main structure.

	Agree	Disagree
Purpose of the tool – This is a tool to facilitate self‐employment for persons with disabilities – Target: the tool targets persons with disabilities (including their characteristics, roles and duties) – Case manager (facilitators): the person or institution equipped to oversee and/or facilitate the steps below, ensuring a structured and effective approach		
Background of the tool – How the tool was developed (3 phases, over 3 years with over 70 participants) – A description of key features (characteristics) to ensure consistency – An outline of legal framework and laws (international and national)		
How to use the tool (ideal tool facilitation) – Outline how to follow the nonsequential Steps 1–4 (process). Emphasise that for effective implementation, there should be tangible progress within 3–6 months, with monitoring for at least a year or more and flexible adjustments as needed – Before Steps 1–4: establish key role players (collaborators) ranging from individuals to institutions, offering services from funding to training (provide a comprehensive list)		
Tool supporting documents – Include easy‐to‐complete and useful checklists and forms, such as the following: – Budgeting template – Business plan form – Community needs assessment checklist – List of small businesses (microenterprises) ∗The user’s guide will be enhanced as the tool matures when it gets tested on the ground		

*Note:* The asterisk means that the user’s guide will be enhanced as the tool matures through real‐world testing; that is, further refinement will occur beyond the scope of this study.

### 2.4. Data Management and Analysis

Microsoft Outlook facilitated effective communication between the researcher, supervisors and participants throughout the data collection process. Microsoft 365 Forms was utilised to design and distribute the online self‐administered questionnaire and efficiently collect participants’ responses. The completed questionnaires were downloaded into an Excel spreadsheet and securely stored online on OneDrive. Only the corresponding author and two coauthors had access to the data.

Following data collection for each round, the Excel spreadsheet was exported and underwent data cleaning, followed by analysis. Data analysis involved thematic analysis by following the Braun and Clarke [29] steps for the qualitative aspects from, for example, Round 1’s open‐ended questions. Descriptive statistics and frequencies, including percentages and means, were used for the quantitative data from, for example, Round 2’s consensus ratings. In line with Helsinki’s Declaration [30], data were coded to reduce bias or ensure participants’ anonymity, with certain columns in the Excel spreadsheet (including demographics, email, province and disability status) concealed. Finally, the analysed data from Rounds 1 and 2 were shared with all participants as part of the subsequent round.

### 2.5. Ethical Consideration

Before commencement, this research obtained formal ethics clearance from the University of KwaZulu‐Natal’s Biomedical Research Ethics Committee (BREC) on 24 January 2023 (ethics number BREC/00004655/2022). All participants received an information sheet and provided written consent to participate. Adhering to core ethical principles, this research prioritised integrity, confidentiality and participants’ anonymity to protect their rights [30].

## 3. Findings

The Findings section is presented under the following subheadings: recruitment and panel selection, Rounds 1, 2 and 3 as outlined under Table5.

**Table 5:** Findings overview.

	Activity	Main findings
Recruitment and panel selection	Bulk mail invitations were sent out	No response
Round 1	Consent received and demographic information collected Response rate *n* = 19/21 (90.47%) Collation of responses to open‐ended questions Round duration: 14 days	Ideal tool considerations and interventions related to entrepreneurial skills for persons with disabilities
		• What (features and components)
		• How (implementation and processes)
		• When and where (key role players and processes)
		• Who (key role players)
		Expected roles and capacities of persons with disabilities

Round 2	Invited *n* = 19 Response rate *n* = 16/19 (= 84.21%) Overall consensus 4.54/5 (90.80%)—ratings using 5‐point Likert scale Round duration: 18 days	• Key features or characteristics of the ideal tool and timelines
		• Legal framework and laws
		• Key role players in using the ideal tool
		• Persons with disabilities’ characteristics, roles and duties
		• Ideal tool facilitation steps (processes), including case management

Round 3 (final)	Invited *n* = 16 Response rate *n* = 15/16 (93.75%) Evaluating a user’s guide with binary agree or disagree options Round duration: 11 days	User’s guide content to help operationalise the framework
		• Purpose of the tool
		• Background of the tool
		• How to use the tool (ideal tool facilitation)
		• Tool supporting resources

### 3.1. Demographics

The participants’ demographics comprised 19 experts, with *n* = 12 females and *n* = 7 males. Representation spanned five out of nine South African provinces, with the majority from Gauteng (*n* = 11), followed by the Western Cape (*n* = 5), and one participant each from the Eastern Cape, KwaZulu‐Natal and Mpumalanga. While their qualifications ranged from Grade 11 to a PhD degree, their average working experience among the participants was 18 years. These participants represented diverse sectors, including government, nongovernment organisations (NGOs), NPOs, private and business settings, healthcare and academia. Refer to Table 6 for the summary of participants’ demographics.

**Table 6:** Summary of participants’ demographics.

	Pseudoname (round completed)	South African province (disability)	Qualification (profession) ∗Master (Master) Master of Science (MSc); occupational therapy (ist) (OT)	Reasons participants are considered research subject experts (for further details, refer to Figure 2: Experts’ experiences and exposure)
1	Hijo (all)	Gauteng	Doctor of Philosophy (PhD) OT, MSc OT and BSc OT (OT)	Owner: 30 years of running my own business, which is a vocational rehabilitation private practice. Researcher: I have supervised students, taken part in postgraduate students’ research and published on work we did at Hillbrow Hospital’s work unit in entrepreneurship for persons with disability. Vocational rehabilitation: The placement in their own businesses has always been an option for service users of our vocational rehabilitation practice. Editor: I have access to publications of the subject matter in (a journal)
2	Joba (all)	Gauteng	MSc OT (OT)	Expert (in vocational rehabilitation and occupational therapy and with business experience)
3	Neta (all)	Gauteng	BSc OT and Postgraduate Diploma in Vocational Rehabilitation (OT)	I have worked in public hospitals with majority of my experience being in the mental health field. I have assessed and treated mental health care users with intervention that focus on return to work as well as their home environments
4	Atoma (Rounds 1 and 2)	Western Cape (quadriplegic)	Diploma in Marketing Management (consultant)	I serve on the board of a few NPOs that represent persons with disabilities. I also have a small business entity
5	Det (all)	Western Cape (tetraplegic C6)	Master of Laws (LLM) and Bachelor of Laws (LLB) (business owner)	My experience in running a small retail specific business is very limited. I am a newbie in selling extra virgin olive oil and other related olive products
6	Roba (Round 1)	Western Cape (stroke)	Grade 11 (business owner)	Experienced business owner
7	Lupa (all)	Western Cape	Doctor of Philosophy (PhD) OT (2005), MSc OT and BSc OT (OT)	Experience as an educator is extensive. Experience as a researcher in entrepreneurship has spanned many years (since late 1990s) but has been secondary to a focus on work and disability. As such, it comprises around 20% of my research focus
8	Jez (all)	Gauteng	Doctor of Philosophy (PhD) OT, M OT and BSc OT (OT)	Occupational therapist with ‘extensive experience in all of these areas’. Refer to Figure 2: Experts experiences and exposure
9	Soso (all)	Mpumalanga (paraplegic)	Grade 12 (business owner)	Experienced business owner and have a good relationship with people in my area
10	Arita (all)	KwaZulu‐Natal	BSc OT and Postgraduate Diploma in Hand Therapy (OT)	I have had positive and negative experiences working in the public health sector. Positive experiences are related to positive outcomes and patient satisfaction. I have had negative experiences also working with some patients, but mostly the negative experiences come from working within rigid structures in public health
11	Eto (all)	Gauteng	Doctor of Philosophy (PhD) (OT)	An experienced occupational therapist who assists patients with mental illness in a private setting. I assist with vocational assessment and vocational interventions in a private mental health setting. I also supervise PhD students at universities
12	Nama (all)	KwaZulu‐Natal	MSc OT, BSc OT and Postgraduate Diploma in Vocational Rehabilitation (OT)	I have experience working at a psychiatric institution under the KZN Department of Health. Here, there were dedicated vocational rehabilitation projects for people with mental health disabilities, with emphasis on community reintegration of MHCUs upon discharge. This included preparation for potential work upon discharge. Furthermore, I was involved in the ‘vocational rehabilitation interest group’ under the KZN OT provincial forum, and chaired the group for a period of 1 year. The interest group gave consideration to vocational rehabilitation resources and practices by OTs across government health establishments within the KZN province
13	Zeba (all)	Gauteng	MSc Medicine and B OT (OT)	Worked as a clinician for 15 years, then as assistant director (AD) Occ Ther in Gauteng Health and deputy director (DD) rehab manager in Gauteng Health and currently rehab manager for FEM in injury on duty and return to work
14	Bela (Round 1)	Gauteng	B OT and Postgraduate Diploma in Vocational Rehabilitation (OT)	I have been working in psychiatry for since 2011, and my focus has been vocational rehabilitation for people with psychiatric conditions. I am currently working at Ekurhuleni Health District (Mental Health Program), and our focus as a team is running income generation projects within the community, as unemployment is a challenge in the district, particularly among mental health care users
15	Nita (all)	Gauteng	Master of Business Administration (Vocational Rehabilitation: Learning and Development)	I have actively being engaged in regulatory framework, where I contributed to the design and development of regulations, protocols, SOPs and policies aimed at supporting persons with disabilities. Additionally, my involvement with vocational rehabilitation has allowed me to support persons with disabilities in their journey to secure and maintain employment through the provision of training and other support services
16	Matu (all)	Western Cape	Doctor of Philosophy (PhD) (OT)	I have extensive experience in providing supported employment services to persons with disabilities and employers
17	Tata (all)	Gauteng (hearing problem)	Master in Public Health (MPH) (OT)	With 10 years of clinical experience as an occupational therapist, I have expertise in patient care, report writing, and management. I have managed the Occupational Therapy Department at Odi District Hospital and the Forensic Occupational Therapy section at Weskoppies Psychiatry Hospital. Since August 2017, I have been a lecturer in the Occupational Therapy Department at (a) University, teaching undergraduate OT students and supervising research. I am also a member of the research committee and am completing my PhD in occupational therapy, with one article submitted for publication in the South African Journal of Education
18	Nora (all)	Eastern Cape	NQF 5 Public Administration (business owner)	Business owner and advance in understanding human nature
19	Toya (Round 1)	Gauteng	Doctor of Philosophy (PhD) OT, M OT and B OT (OT)	Have worked in VR for many years in all sectors. I was involved in MODE as a director and was actively engaged in small business development in many settings, with a focus on PWDs

**Figure 1:**
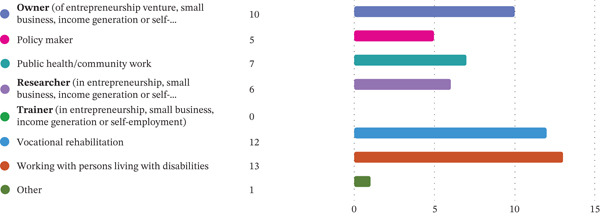
Participants’ experiences and exposure.

**Figure 2:**
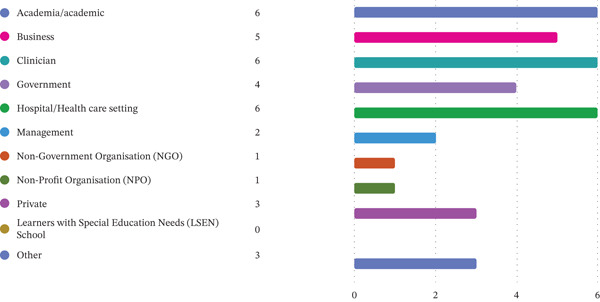
Participants’ institution, designation or sector descriptors.

In terms of disability representation, six participants (*n* = 6) identified as persons with disabilities, comprising one sensory disability and five physical disabilities. The participants included interdisciplinary professionals, including occupational therapists from various settings, Department of Employment and Labour (DoEL) representatives, business owners with disabilities and representatives from persons with disabilities organisations, that is, end users, service providers and policy actors. Refer to Figure 1 for participants’ experiences and Figure 2 for the participants’ institutional affiliations, designations and sector descriptors.

#### 3.2. Rounds 1–3 Findings

##### 3.2.1. Round 1 Findings

In Round 1, 19 out of 21 participants (90%) completed the survey, with an average completion time of 53 min and 36 s over 14 days. The majority (*n* = 13, 68%) were unaware of tools intended to assist persons with disabilities in self‐employment. While *n* = 3 (16%) were unsure, *n* = 3 (16%) were aware of such tools. They were aware of tools encompassing learnerships, vocational education programmes, models and South Africa’s enabling laws and policies. The main findings of Round 1 are reported under the following themes: (i) ideal tool considerations and interventions related to entrepreneurial skills for persons with disabilities (the participants focused on the *what*, *how*, *when* and *where* and *who* to be considered or included) and (ii) expected roles and capacities of persons with disabilities.

###### 3.2.1.1. Ideal Tool Considerations and Interventions Related to Entrepreneurial Skills for Persons With Disabilities

What (features and components of the ideal tool): The participants reported that the ideal tool for facilitating self‐employment among persons with disabilities should be a comprehensive, accessible tool that addresses the unique needs of persons with disabilities. They added that the ideal tool should include skills identification, business and vocational training, financial management, marketing and product knowledge, focusing on entrepreneurship and self‐employment opportunities. Moreover, they stated that the tool should be contextual, tailored to persons with abilities and consider micro to macro factors, including environmental and cultural sensitivity. The participants believe the tool should guide persons with disabilities through all stages of business development, from idea generation to registration, funding applications and upskilling, with easy‐to‐answer questions and a user‐friendly interface. Furthermore, they stated that the tool should prioritise sustainability, long‐term support and collaborative partnerships to ensure persons with disabilities have targeted policy support and access to crucial resources. Below are some important quotes from the participants:

*“Focus must be on all aspects related to skill development of the whole business”*. (Toya)

*Overall: It* [tool] *should guide and inform the practice of occupational therapists to encourage and enable their clients to start and successfully run their own business*. (Hijo)

*“I think that if the guideline provides sufficient detailed information, checklists and steps, it will be a HUGE asset… The guideline/training program should be detailed enough for a newly qualified therapist to follow, as many health institutions have only CSO therapists* (community service occupational therapists) *yearly”*. (Nama)

How (implementation and processes of the ideal tool): According to the participants, the implementation of the ideal tool for facilitating self‐employment among persons with disabilities should follow a phased, staged, structured and hybrid approach, combining online and offline components. They reported that these could include stages such as pretraining, implementation and follow‐up monitoring and evaluation. While the participant stated that hands‐on incubation training should focus on holistic upskilling in business and life skills, they emphasised customisation of the tool to ensure no one‐size‐fits‐all approach by considering injury history, capabilities and contextual factors. They concluded by reporting that remote support elements should accommodate resource constraints, ensuring sustainability and success. The following are important quotes from the participants:

[The tool should have] “*PREP and TRAINING phase. IMPLEMENTATION, FOLLOW UP, MONITORING AND EVALUATION phases”*. (Toya)

*“Ensure success (i.e. using elements of the “place and train” approach… PWD [should not be] left unsupported in the community… Use of Adult learning principles.”* (Nama)

When and where: The participants reported that information and resources about the ideal tool for facilitating self‐employment among persons with disabilities should remain accessible in community settings and be implemented at any point in the ecosystem or integrated into existing systems. They noted that the easily accessible settings to persons with disabilities should include clinics, community centres, hospitals, NGOs, NPOs, rehabilitation centres and schools. As Arita stated, *“A tool that will address the needs of PWDs* [persons with disabilities] *in rural and urban centres* [is needed]*”.*

Who (key role players): According to the participants, facilitating self‐employment among persons with disabilities requires a multidisciplinary team approach, involving collaboration between various key role players, including persons with disabilities, their families and guardians, therapists, public authorities or advisors. The participants reported that a referral system between those involved should be established to ensure seamless transitions and coordination so they can leverage existing resources and avoid duplication of efforts. Below is an important supporting quote from a participant:

*“I think OTs* [occupational therapists] *should lead, potentially wearing two caps, i.e. of a clinician and case manager, to ensure that services take place. While major cities in SA [South Africa] do have many organizations such as NGOs, NPOs, etc to offer support, there are less opportunities like this in smaller cities and towns in South Africa. Therefore in my humble opinion, in public-health cases in areas with limited external resources, it is left to the OT to facilitate and drive the RTW [return to work process] and self-employment venture of the client, which includes teaching basic entrepreneurial skills. For this reason, I think OTs should lead to ensure sustainability of the guideline. Referrals can be made to other health professionals, learnerships, NPOs and NGOs as needed (recommendations could be included in the guideline)*”. (Nama)

##### 3.2.1.2. Expected Roles and Capacities of Persons With Disabilities

The participants indicated that there should be no restrictions on the capacity in which persons with disabilities participate in self‐employment, emphasising the importance of being proactive and entrepreneurial. In addition to other expectations, they believe that persons with disabilities could serve as business experts, inform policy decisions and engage in role modelling and mentoring to support other persons with disabilities in self‐employment. According to the participants, some examples of capacities that accommodate individual preferences could include owner (founder/employer), co‐owner, manager and trainer. Below are some comments to support the above:

*“There needs to be motivation from the participant”*. (Neta)

*“Persons with disabilities should be owners, co-owners, managers, trainers, and employers and any other capacity expected from any entrepreneur”*. (Matu)

“[Persons with disabilities should be involved in] *All aspects. Nothing without the full support, input and collaboration of PWDs… The business can only flourish to the level that the PWD can see it and which is congruent to how they see SELF”*. (Toya)

#### 3.2.2. Round 2 Findings and Group Consensus

In Round 2, 16 out of 19 participants (84.21%) submitted responses, with an average completion time of 38 min over 18 days. Overall consensus for Round 2 was 4.54/5 (90.80%). The Round 2 main items are listed below and detailed in Table 7 which include the *Tool timelines*, *Key features or characteristics of the ideal tool*, *Legal framework and laws*, *Key role players in using the ideal tool*, *Persons with disabilities’ characteristics, roles and duties* and *Ideal tool facilitation steps (processes)*—including case management.

**Table 7:** Round 2 findings and group consensus.

Section		Description	Section average score/5	Section average %
Section A: Key features or characteristics of the ideal tool		Sustainable (not short‐lived or once‐off), for example, be meaningful and impact persons with disabilities lives	4.54	90.83
		Clear or transparent and straightforward, for example, by following result‐driven steps to track progress		
		Accessible, for example, outreach‐oriented (bringing services closer to persons with disabilities) or within reach to persons with disabilities such as face‐to‐face in their communities and/or exploring online services		
		Adaptable, contextual, nonprescriptive and responsive, for example, cater for community (rural/urban) specific needs yet able to evolve and leverage or use technology		
		Practical, realistic and tailored (individualised), for example, within persons with disabilities interests and/or abilities—no blanket approach		
		Allow or facilitate access to business finance, material, equipment, tools and assistive devices and market		

Section B: Legal framework and laws	International legal frameworks or laws	The Declaration of the Alma‐Ata encourages collaboration beyond the health sector to ensure the sustainability of primary health care, which is for economic development through, for example, the running of successful microenterprise by persons with disabilities	4.36	87.20
		The World Health Organisation (community‐based rehabilitation [CBR] livelihood components) states that CBR programmes have an integral role to play in helping people with disabilities become self‐ employed by starting or expanding their own income‐generating activities and small businesses		
		The International Labour Organisation (ILO) acknowledges the importance of work or vocation, including for persons with disabilities. As an ILO signatory, South African legislature and practices should adhere to agreements and ensure persons with disabilities are fully supported in their microenterprises		
	National (South African) legal frameworks or laws	Preferential Procurement Policy Framework Act 2000 (PPPFA) states that a preference points system should be used to give preference, for example, government spending or contracts toward previously disadvantaged populations		
		Promotion of Equality and Prevention of Unfair Discrimination Act 4 of 2000 (PEPUDA), in line with the South African Constitution, prohibits discrimination on the grounds of disability, such as failing to eliminate obstacles that unfairly limit or restrict persons with disabilities from running a microenterprise, for example, lack of access to suitable training		
		The National Development Plan 2030 (NDP) encourages citizens to be active in their own development and to raise economic growth		

Section C: Key role players in using the ideal tool		Business and private individuals or institutions, for example, business experts offering business or legal pro bono services	4.45	89.11
		Consumers or customers who, for example, buy products and use services offered by persons with disabilities in their microenterprise		
		Family members or relatives offering support, for example, personal and physical support or assistance in running the microenterprise		
		A multidisciplinary team (MDT), for example, rehabilitation staff addressing needs such as vocational, mental and physical health		
		Nongovernment organisations (NGO) and nonprofit organisations (NPO), for example, Disabled People South Africa (DPSA) or QuadPara Association of South Africa (QASA), which advocates for persons with disabilities needs		
		Persons with disabilities, for example, as role modelling and providing mentorship or training to other persons with disabilities		
		Government or public institutions, for example, the Department of Employment and Labour (DoEL), Department of Planning Monitoring and Evaluation (DPME), Department of Small Business Development (DSBD), Department of Women, Youth and Persons with Disabilities (DWYP) and Small Enterprise Development Agency (SEDA) as they align with the South African Constitutions, which is pro persons with disabilities needs		
Section D: Persons with disabilities’ characteristics, roles and duties		Have a sense of and positive view of self	4.40	88.11
		Be willing to be or be the primary driver of the business		
		Be motivated and have the desire to be self‐employed		
		Show proactiveness by making plans and acting on them		
		Ability to network with other key role players and mentor		
		Persons with disabilities’ roles and duties in the business should not be limited but in keeping with their abilities and/or interests, for example, sole or co microenterprise owner and employer or manager		
		Other duties and expectations could be give back (by, e.g., mentorship and role modelling), training others, (e.g., in the form of mentorship and apprenticeship) or being active by informing relevant policies		

Section E: Ideal tool facilitation steps (processes)	Step 1	Identification and profiling of community and outside community resources, for example, make a list of key role players and initiatives such as available microenterprises	4.18	83.75
		Identification and recruitment of suitable persons with disabilities, for example, using existing platforms such as health care settings, schools, church and community business forums		
		Profiling persons with disabilities’ abilities, for example, level of function, microenterprise interests or assessing their work abilities/abilities to own a microenterprise		
	Step 2	Match persons with disabilities to a suitable microenterprise, for example, retail (buy and sell), rendering a service and manufacturing/production	4.64	92.92
		Facilitate training (upskill) and educate through, for example, place and train approach, apprenticeship or incubation		
		Assist with business financing, for example, application process, securing and management of a grant		
	Step 3	Microenterprise placement, for example, by setting up a new or building on an existing microenterprise—securing tools, material, site and human resources	4.56	91.25
		Running the microenterprise, for example, opening the microenterprise daily, making sales, transacting and closing the microenterprise daily		
	Step 4	Follow up by, for example, offering ongoing support through coaching and mentoring	4.78	95.63
		Monitor, revaluate and repeat Steps 1, 2 and 3 or terminate the process		

**Graded four steps**	Case manager	This graded four steps to succeed need an individual or institution to oversee (case manager) and/or facilitate	4.37	87.50

##### 3.2.2.1. Tool Timelines

The participants provided guidance on the implementation timeline for the tool components and overall tool, suggesting that implementation should commence within 3 months. They cautioned against undue delays, stressing that visible or tangible results are essential. Furthermore, the participants emphasised the critical importance of ongoing support and a customised, case‐by‐case approach, tailoring the tool’s application to individual needs. To ensure effective implementation, the participants recommended demonstrating tangible progress within 3–6 months of self‐employment facilitation and monitoring for at least a year or more, with flexible adjustments. In this context, timelines assist with facilitation processes, prioritisation and sequencing, thereby supporting the framework’s feasibility. Nama concluded by saying, “*It* [the tool] *could take a minimum of 6 months but up to 10 months to a year depending on the complexity of the case*”.

##### 3.2.2.2. Section A: Key Features or Characteristics of the Ideal Tool

The participants concurred that, for consistency, the ideal tool should possess and adhere to clear, essential features while being as explicit and detailed as possible. Their average agreement rating is 4.54/5 (90.80%). Neta stated, *“The tool should not be too lengthy. It should allow for necessary rehabilitation, however, consider changing markets. The outcome should be clear”.*

##### 3.2.2.3. Section B: Legal Framework and Laws

The participants agreed (4.36/5, 87.20%) that the ideal tool facilitating self‐employment should align with relevant international and national (South African) legal frameworks or laws. However, they expressed concerns about the effectiveness and implementation of existing laws, recommending a closer examination and checking the effectiveness of global standards such as the Declaration of Alma‐Ata and the International Labour Organisation (ILO), as well as South African policies like the Preferential Procurement Policy Framework Act (PPPFA) 2000 and the National Development Plan (NDP) 2030.

##### 3.2.2.4. Section C: Key Role Players in Using the Ideal Tool

The participants agreed (4.46/5, 89.1%) that the nonprescriptive ideal tool requires collaborative efforts from credible and reliable key role players across various settings, from community to national and international levels, to effectively support persons with disabilities in self‐employment, that is, the collaborations should transcend siloed approaches. Neta reported, *“For sustainability of the tool there needs to be buy in particularly from private partnerships and government entities. Citizens, families and NGOs are likely to support the tool and outcomes when they can see buy in from other stakeholders”.*

##### 3.2.2.5. Section D: Persons With Disabilities’ Characteristics, Roles and Duties

The participants reached a consensus (4.41/5, 88.20%) that the nonprescriptive ideal tool should prioritise capable persons with disabilities and position (place) them in self‐employment initiatives. Furthermore, they agreed that the tool should clearly define roles and duties, primarily focusing on empowering individuals with disabilities to drive their entrepreneurial ventures rather than solely supporting others. Zeba concluded by saying, *“A person has to be self-driven”.*

##### 3.2.2.6. Section E: Ideal Tool Facilitation Steps (Processes)

The agreement among participants averaged 4.54/5 (90.80%) regarding the importance of following a structured process (steps) when mobilising resources and facilitating self‐employment for persons with disabilities using the nonprescriptive ideal tool. They emphasised that the sequential steps may overlap or intersect, requiring flexibility and adaptability in their application. The steps they suggested are as follows: Step 1 (preparing the ground or conducting a situation analysis), Step 2 (intervention or bridging the gap), Step 3 (placement or setting up the microenterprise) and Step 4 (carryover and stabilising the microenterprise). Lupa stated that *“These steps might be conceptually separate, but practically, they are merged”* and was supported by Zeba, who said that *“I think many of them* [steps] *can run concurrently”.*

The participants agreed (4.375/5, 87.5%) that successfully implementing the graded four steps requires dedicated support and guidance to succeed, that is, a case manager. They supported the need for an individual (case manager/facilitator) or an institution to oversee and/or facilitate these steps, ensuring a structured and practical approach. While Zeba suggested that *“Involvement* [of a case manager or facilitator] *will need to be worked out on a case by case basis”.* Other participants submitted that the case manager needs some knowledge to guide the process.

#### 3.2.3. Round 3 Findings

Round 3 had 15 out of the 16 participants invited, that is, 93.75% response rate. The average time for this round was 25 min (over 11 days), that is, the shortest round. The overall consensus for Round 3 was 98.33%. The findings of Round 3 show what the participants agreed should be included in a friendly user’s guide to operationalise the tool or framework. The agreed‐upon considerations for inclusion in the user’s guide are as follows: (i) *purpose of the tool*, (ii) *background of the tool*, (iii) *how to use the tool (ideal tool facilitation)* and (iv) *tool supporting resources*.

The participants reported that the tool’s purpose had to be explicit on what it is intended for, the types of impairment it targets and for use by which profession/al, for example, an occupational therapist. They added that some terms, including ‘tool’ and ‘characteristics’, need to be better defined for effective operationalisation. The aspects of background (development methodology) and on how to use the tool received similar comments, as the participants stated that they too should be explicit. On the tool supporting resources, the participants provided enhancement suggestions, including the addition of a resource list (e.g., professionals and institutions) and a question template for user reference, as summarised by the following quote from Det, who stated that *“Perhaps it* [the tool] *should also include a list of professionals, like bookkeepers and accountants, that have been vetted and approved. It should also have a link to government resources that are readily available, like SEDA etc… I suggest* [having] *a question template similar to the SARS e-filing [South African Revenue Service online tax return system] approach, where the questions answered generate the template document either to be completed or already completed. So, each answer either results in more questions or closes an option out”.* (Det).

Further suggestions on operationalising the tool, the participants recommended that the facilitating professional should be involved in every step of the process and meet predetermined criteria or training, that is, a case manager. Furthermore, they emphasised the importance of piloting the tool in diverse settings or fields of practice to solicit further feedback and critique. Ultimately, the participants concluded that the tool should cater to both physical and mental needs, that is, one of its purposes. Nama summed it up by saying that there should be a consideration of *“…Introducing the tool to occupational therapists in various settings such as hospitals, PHC clinics, private practices through CPD events, allowing them to try it in their settings with opportunities to provide feedback on the operational aspect from different perspectives (relative to the setting, socioeconomic status of clients). Also, introducing the tool to OT departments in higher education institutions across SA* [South Africa] *for consideration during student training”.*

In closing, the participants shared that for future consideration, the following two key enhancements should be taken into account: developing a mobile application and translating or making the tool available in different South African languages for accessibility and usability. Neta stated that *“It would be awesome if in the future the tool can be developed on an app… This could then be updated with new information and resources as it becomes available”.*

## 4. Discussion

The discussion addresses three primary areas aligned with this research’s focus and supported by existing literature: methodological rigour, the agreed‐upon ideal framework components and operationalisation considerations for implementation.

This research, part of a multiphase PhD project, employed a systematic and rigorous approach to develop components of an evidence‐based framework to support persons with disabilities in self‐employment. A scoping review (Phase 1) provided the evidence base, semistructured interviews with key role players (Phase 2) identified context‐specific needs and a Delphi study (phase three) iteratively built expert consensus on the framework’s essential components. The Delphi technique’s established credibility and rigour [23, 31] were enhanced by the diverse interdisciplinary sample representing varied institutional affiliations, designations and sectors. The participants’ extensive average experience of 18 years strengthened the validity and reliability of findings, while the high response rates (Round 1: 90.47%, Round 2: 84.21% and Round 3: 93.75%) demonstrated strong engagement and commitment to the research process.

The Delphi process commenced with an initial pool of 21 participants and concluded with 16 participants. Attrition occurred as some participants did not respond to follow‐up emails; this is a common phenomenon in Delphi studies. As such, caution should be exercised when interpreting these findings. They should serve as evidence‐based guidance rather than generalisable prescriptions, with contextual adaptation recommended for implementation. Refer to the recommendations below for more information. Importantly, the panel retained disciplinary and sectoral diversity across rounds, and their input (the framework’s components) was strengthened through triangulation with the literature, supporting methodological rigour.

Before rating the framework (tool) components in this research, the participants gave an overview of what the framework should encompass, which entailed the ‘what, how, when and where and who’. Their responses indicated support for developing such a framework.

Potential barriers to operationalisation of the framework were highlighted early in the Delphi process, with participants highlighting the staff shortages, which would exacerbate the lack of knowledge and interest in implementing the final framework. This aligns with the budgeting trend where costs are cut, observed in South African government spending [32, 33], which affects the already low number of healthcare professionals, including occupational therapists [33]. Adding to the above is the observed lack of interest in this field [6] despite professionals involved in vocational rehabilitation [11, 12]. Difficulty in implementing the framework may be further exacerbated by the time‐consuming facilitation process proposed by the participants in this research, as it could take up to a year to set up the business with a person with a disability.

Participants emphasised operationalisation considerations emphasising that it is critical for a person with a disability to make tangible progress in establishing the business within 3–6 months to at least a year. These comments align with findings by Alexander et al. [15] who stated that time can be a significant factor in microenterprise processes, where 4 years was the minimum their participants took to set up their businesses. This encompassed activities such as sourcing funding, training participants and setting up the microenterprises. These researchers believe 4 years is necessary for the entire process to be covered effectively. They also added that certain parts, including sourcing funding, can be addressed ahead of the whole process [15].

The framework’s nonprescriptive nature emerged as a fundamental characteristic, allowing adaptability across diverse contexts. In the discussion of its potential application, participants referred to variations in rural, urban and cross‐border applications within the African region. These references reflect how participants naturally contextualised the framework within their practice environments, rather than suggesting that its use is confined to these regions. One participant contextualised the framework within their reality, that is, ‘Think wider than just South Africa ‐ we have a lot to learn from and to offer our neighbouring/other African countries. OT [occupational therapy] clients are… Often from Lesotho, Zimbabwe, Namibia, Malawi, etc.’ (Hijo). This perspective demonstrates how the framework can accommodate transnational diversity while still aligning with its intended applicability.

The reflections above underscore the need for flexibility and adaptability to contextualisation within the framework. Alexander et al. [15] emphasised the need for transparency and contextualisation, which can be implemented through mechanisms such as a comprehensive checklist and detailed guidelines. Thus, the framework should provide a clear and transparent process that can be followed by all facilitators or case managers, including novice practitioners, while still ensuring tailored interventions for persons with disabilities. This approach supports the development of a framework that is both globally relevant and locally responsive. In this sense, the developed framework should act as a guide, while practitioners customise its application based on individual needs and the specific micro and macro contextual factors within which clients function.

In line with Sodhi and Dwivedi [6], who call for continued research, and Shafaghat et al. [20], who emphasise the role of scientific evidence in decision‐making, the importance of this study’s findings is highlighted by the strong consensus among participants on the framework’s essential components. The key components and ratings include (i) *key features* (90.8%), which are crucial to ensure consistency when implementing the framework. Moreover, (ii) *legislation* (87.3%) was perceived as essential, incorporating both international (global) laws that inform national disability rights legislation. At the global stage, the participants highlighted laws from organisations that South Africa is a signatory to, including the United Nations. Yet, they warned that some statutes or their impact do not filter down to the local level for inclusiveness or promoting disability rights as they claim. This concern is consistent with literature noting gaps between disability‐related policies and their implementation in practice [34, 35]. Their warnings about implementation gaps between international declarations and local practice highlight persistent challenges in disability rights actualisation. The participants are adamant that the framework should be able to bring services and resources to persons with disabilities [40, 51]. The emphasis on bringing services directly to persons with disabilities addresses these structural barriers while leveraging existing legal protections. Moreover, they are prointroducing resources, mobilising existing ones or enhancing or repurposing them for better use and access. Agreement among the participants was also reached on collaborative efforts, discouraging silo work and identifying (iii) *key role players* (89.1%), including credible and empowered persons with disabilities, caregivers, community initiatives and healthcare professionals. A similar emphasis on improving access to services and mobilising resources for persons with disabilities is reflected in existing literature [6, 34, 35]. This suggests a need for an interdisciplinary and multidisciplinary team [6] with a viable and effective referral system to benefit persons with disabilities in self‐employment. This multistakeholder approach aligns with community‐based rehabilitation principles and addresses the complexity of barriers facing persons with disabilities in employment contexts. The consensus on (iv) *characteristics, roles and duties of persons with disabilities* (88.1%), emphasises that persons with disabilities should not be limited but be involved in business like anyone or able‐bodied individuals, but ensuring that their role matches their abilities [36]. Moreover, they emphasise capability over limitation, positioning individuals with disabilities as primary drivers of their entrepreneurial ventures, a notion supported by empowerment‐focused studies [37, 38]. The framework’s emphasis on mentoring and role modelling creates pathways for peer support and knowledge transfer within disability. Lastly, they agreed on the ideal tool’s (v) *facilitation steps* (90.9%) that should be followed, not rigidly, but to be used as a guide. An approach was supported by Alexander et al. [15], who advocate for flexibility to foster creativity. These participants’ emphasis on an explicit user’s guide aligns with the need to close the gap, such as the lack of profession‐specific frameworks in this field [5]. Furthermore, the focus on operational clarity and validation of the guide to enhance its practical application aligns with the phased research approach and theoretical integration proposed by Alexander et al. [15] and Sodhi and Dwivedi [6]. Ultimately, this user’s guide has the potential to translate expert consensus and available literature into an actionable tool, making it an evidence‐based methodology, as supported by Shafaghat et al. [20].

## 5. Conclusion and Recommendation

This multiround Delphi study successfully established participants’ consensus on components of an evidence‐based framework for facilitating self‐employment among persons with disabilities. The participants gave an overview of what the framework should encompass, which entailed the ‘what, how, when and where and who’ of the framework. They collectively emphasised that the prospective framework should be nonprescriptive, prioritising practicality, accessibility, implementation and sustainability within and beyond the South African context.

The limitations in this study were (i) small panel size and (ii) attrition which may affect transferability. Participants proposed measures like a user’s guide to support framework operationalisation. Thus, the final deliverable operationalises the framework, enabling those involved to support persons with disabilities in pursuing self‐employment for independent work. The development of a comprehensive user guide emerges as the critical next step for operationalising the framework. This guide should include detailed protocols, assessment tools, resource directories and outcome measures to address identified barriers and ensure consistent implementation across settings.

The rated components should be synthesised and enhanced to further develop this research’s findings. This step will ensure a robust and effective evidence‐based framework for facilitating self‐employment for persons with disabilities. This can be done by utilising theories such as empowerment theory and system theory. The empowerment theory will highlight the framework’s emphasis on promoting persons with disabilities’ rights, autonomy and self‐determination. On the other hand, the system theory will guide the complex and dynamic interactions of the framework’s five key components from a macro to a micro level, that is, between individuals, organisations and systems. In addition to the user’s guide, further considerations are necessary to operationalise the framework. These include developing a comprehensive list of possible microenterprises suitable for persons with disabilities. A systematic approach to selecting and analysing these microenterprises is also crucial, taking into account individual skills, interests and abilities. Furthermore, a detailed list of key role players, their specific roles and responsibilities will be essential to ensure effective implementation and ongoing support.

## Author Contributions

Mr. Luther Lebogang Monareng was responsible for heading the project, conceptualising and drafting the manuscript. Professor Shaheed Mogammad Soeker and Professor Deshini Naidoo assisted with the conceptualisation of the manuscript, guidance and critical reviews throughout the writing process of the manuscript.

## Funding

The study was supported by (i) South Africa’s National Research Foundation (NRF), (ii) University Capacity Development Grant Funding (UCDP) from the University of KwaZulu‐Natal and (iii) the University of KwaZulu‐Natal’s Step Up Programme, Department of Higher Education and Training (DHET) and Newton Fund.

## Ethics Statement

Before commencement, this research obtained formal ethics clearance from the University of KwaZulu‐Natal’s Biomedical Research Ethics Committee (BREC) on 24 January 2023 (ethics number BREC/00004655/2022). All participants received an information sheet and provided written consent to participate. Adhering to core ethical principles, this research prioritised integrity, confidentiality and participants’ anonymity to protect their rights [30].

## Consent

The recruitment email included an information sheet, consent form, participant expectations and an ethics clearance letter.

## Conflicts of Interest

The authors declare no conflicts of interest.

## Data Availability

The data that support the findings of this study are available from the corresponding author upon reasonable request.
